# Hepatitis B vaccination status and associated factors among undergraduate students of Makerere University College of Health Sciences

**DOI:** 10.1371/journal.pone.0214732

**Published:** 2019-04-05

**Authors:** Yvette Wibabara, Cecily Banura, Joan Kalyango, Charles Karamagi, Alex Kityamuwesi, Winfred Christine Amia, Ponsiano Ocama

**Affiliations:** 1 Clinical Epidemiology Unit, Makerere University, Kampala, Uganda; 2 Department of Paediatrics, Makerere University, Kampala, Uganda; 3 Department of Medicine, Makerere University, Kampala, Uganda; Centre de Recherche en Cancerologie de Lyon, FRANCE

## Abstract

**Background:**

Hepatitis B is a global health problem. Trainees in the health-related fields are exposed to occupational risk of Hepatitis B Virus. In Uganda, there is scarcity of information on vaccination among students in health-care. The objective of this study was to assess hepatitis B vaccination status of the students and factors associated.

**Methods and findings:**

This was a cross sectional study, conducted at Makerere University College of Health Sciences among undergraduate students who were eligible. A self-report on Hepatitis B vaccination status and various characteristics were collected on each participant, using a standardized structured self-administered questionnaire. Descriptive statistics were computed, bivariate and multivariate analysis were done using Stata 14.

**Results:**

Out of 760 participants, 44.3% (95% CI 35.2–52.8) reported full vaccination. Vaccination was associated with gender, course, year of study and student’s sponsorship. Males were less likely to be vaccinated, Prevalence Ratio (PR) 0.79; P-value <0.001, while self-sponsored students were also most likely to be vaccinated, PR 2.08; P-value <0.001. About 37% reported an accidental needle injury during their training.

**Conclusion:**

Full vaccination was low and given the high prevalence of needle injuries, it raises a safety concern. Vaccination should be mandatory for all students prior to clinical exposure. There is need for targeted interventions to increase uptake.

## Introduction

Hepatitis B is a viral infection that attacks the liver. It is a major global health problem, and the most serious type of viral hepatitis. It is estimated that about 780,000 people die each year due to consequences of hepatitis B [1]. In Uganda, Hepatitis B infection is highly endemic with a national prevalence of 10%, with geographical variation across the country ranging from 4% in the southwest 5% in Kampala and surrounding districts and 25% in Northeast [2].

Like other health workers, trainees in the health care professions are also exposed to an equal magnitude of occupational risk of Hepatitis B Virus (HBV), as they work in the same health care delivery system. In fact, the risk for accidental exposure among the trainees could be higher due to their lack of experience and insufficient training on how to use Personal Protective Equipment PPE [3]. A study done among medical students in School of Medicine Makerere University Uganda found that overall 11% of medical students had hepatitis B infection defined as Hepatitis B surface antigen (HBsAg) positive[4].

Uganda’s endemicity for Hepatitis B infection poses a great risk of occupational exposure to HBV for persons in health-related fields. World Health Organization (WHO) guidelines for the prevention, care and treatment of hepatitis B infection recommend special consideration of health-care workers and students for HBV screening and vaccination. However, these guidelines are not widely implemented in Low and Middle-Income Countries [5]. The Uganda Ministry of Health’s statutory instrument on hepatitis B requires all health workers and students to be vaccinated against HBV within six months from the commencement of clinical exposure.

There is paucity of information about what the current Hepatitis B vaccination coverage among students in the health-related fields. Therefore, this study sought to assess the vaccination status of students in the College of Health Sciences and the factors associated. The study findings will generate information necessary for formation of policies on vaccination against Hepatitis B among students in health related fields.

## Materials and methods

This was a cross sectional study conducted at Makerere University College of Health Sciences (MakCHS) from March- April 2018. Makerere is the biggest University in Uganda located in Kampala district in Central region. Makerere has 9 colleges, one of them being College of Health Sciences (CHS). The college admits students for various courses including: Medicine (MBChB), Nursing (NUR), Pharmacy (BPH), Radiography (BMR), Biomedical sciences (BSB) Environmental Health (BEH) and Dental surgery (BDS). The students are attached to Mulago hospital which is a National Referral Hospitals also acting as the teaching hospital for the undergraduate and graduate students for their clinical rotations. Students in BSB undergo training for 3 years, NUR, BPH and BMR train for 4 years, while MBChB and BDS train for 5 years.

### Study population

The study involved all undergraduate students in the MakCHS that were in the study area during the study period who fulfilled the eligibility criteria. We included all consenting students enrolled in MBChB, BDS, BPH, BMR, BSB and NUR who were found in lecture rooms during the study period and excluded anyone with a known diagnosis of hepatitis B infection based on a self-report.

### Ethics approval and consent to participate

Permission to conduct the study was sought from the Clinical Epidemiology Unit, MakCHS. Institutional ethical approval was sought from Makerere University School of Medicine Research and Ethics Committee (SOMREC). Approval was granted ref. no. REC REF 2018–047 Administrative permission to conduct the study was granted by the college principal. All participants were requested to consent before participating in the study. Confidentiality of participant information was ensured by not using participants ‘names on the questionnaires.

### Sample size

**Objective 1** Using Kish Leslie 1965 formula for a descriptive objective, the proportion of vaccinated students, from literature being 0.39 [6] the required sample size was 365. But since by nature of the study there was likely to be design effect we adjusted the sample size, using a design effect of 2, the adjusted sample size to 730 participants and anticipating some none response, we added 10% making it 803.

### Sampling

We planned to use proportionate stratified random sampling. The study population was to be stratified on course offered and a proportionate random sample selected from each stratum. However, during data collection, the turn up of students for lectures was low, so we enrolled everyone who fulfilled the eligibility criteria.

### Variables

**Dependent variable**: Vaccinated, coded as 1 “Yes” 0 “No”. All those who had received three or more doses were coded as “Yes” while those who had received less than three doses, or never been vaccinated or not sure of their vaccination status were coded “No”

**Independent variables**: Age category, sex, year of study, course, sponsorship, source of upkeep, religion, marital status, nationality, knowledge category, risk perception, history of needle prick injury. Knowledge was categorized into “knowledgeable” if the respondents were able to answer 70% or more of knowledge questions correctly. “less knowledgeable” if the respondents answered less than 70% of knowledge items. The tool for assessment on knowledge had questions on transmission of hepatitis B, complications and prevention.

### Data collection and management

Data collection was done at opportune moments when students had come together for lectures. The pretested self-administered questionnaires were distributed to all students and collected immediately to minimize none response. Students who did not consent and those who had been diagnosed with Hepatitis B returned both the unfilled questionnaire and consent form. Data was entered into Epidata version 4.2. The raw data was first exported to excel, cleaned and later imported into STATA version 14.0. for analysis.

### Analysis

The demographic characteristics were described using proportions for categorical data, median and inter-quartile range for continuous variables. The proportion describing the vaccination status of participants was computed. Note that because the outcome was not rare (>20%) a logistic model was not appropriate for analysis. So, we used a modified Poisson model reporting clustered robust standard errors, significant variables were checked for interaction, dropped variables were tested for confounding.

## Results and discussion

Out of 820 students that received the questionnaires, 760 (93%) responded, 9 (1%) fulfilled the exclusion criteria of having Hepatitis B infection, while 51 (6%) did not respond. The median age of the 760 participants was 23, range (18–42) years. As shown in Table 1, majority were (65.1%) were males, while about 50.8% (386/760) were MBChB students.

**Table 1:** Socio-demographic characteristics of the 760 study participants at MakCHS March- April 2018.

Variable	Category	Number (n = 760)	Percentage (%)
Sex	Female	265	34.9
	Male	495	65.1
Age category	18–30	715	94
	31–49	45	6
Course	BMR	62	8.2
	NUR	61	8
	BSB	54	7.1
	BDS	87	11.4
	BPH	110	14.5
	MBChB	386	50.8
Year of study	I	176	23.2
	II	148	19.5
	III	186	24.5
	IV	147	19.3
	V	103	13.5
Marital status	Married	72	9.5
	Single	631	83
	Others*	57	7.5
Religion	Christian	658	86.6
	Moslem	73	9.6
	Others^	29	3.8
Nationality	Ugandan	715	94.1
	Non-Ugandan	45	5.9
Region of origin (n = 715)	Central	321	44.9
	Western	164	22.9
	Eastern	146	20.4
	Northern	84	11.7
Sponsorship	Government	414	54.5
	Parents/guardian	276	36.3
	Private sponsorship	66	8.7
	Self	4	0.5
Student upkeep	Parents	452	59.5
	Guardian	52	6.8
	Sponsor	42	5.5
	Self	197	25.9
	Spouse	17	2.2

*1 cohabiting, widowed, divorced

^2 Asian Religions

### Hepatitis B vaccination status and associated factors

Of the 760 participants, 44.3% (337/760) reported having received all the three doses required for protection against HBV, 22.5% (171/760) reported partial vaccination, while 33.2% (252/760) had not received any vaccination against HBV. Of those who were not vaccinated (239/760), 63.2% stated high cost of vaccine as the most common reason hindering their vaccination, 9.5% believed they were not at risk while 27% did not know where to find the vaccine. 74.6% of the participants were knowledgeable about hepatitis B infection. Most of the participants (67.8%) perceived themselves to be at high risk of occupational exposure to hepatitis B, in addition, 36.7% (279/760) reported having had a needle stick injury while attending to patients and of these 19.0% (53/279) had taken PEP for HIV. Of those who reported needle stick injuries, 50% (50/279) were in their last year of training.

As shown in Table 2 as well as Table 3, vaccination was significantly associated with sex, course, year of study and student’s sponsorship.

**Table 2:** Bivariate analysis of the association between social demographic characteristics and Hepatitis B vaccination status.

Variable	Number vaccinated (%)	Number not vaccinated (%)	Prevalence ratio	95% CI	P-value
**Sex**					
Female	134 (50.6)	131 (49.4)	1		
Male	203 (41.0)	292 (58.9)	0.81	0.68–0.96	0.016
**Age category**					
18–30	318 (44.5)	397 (55.5)	1		
31–49	19 (42.2)	26 (57.8)	0.94	0.62–1.44	0.808
**Course**					
BMR	20 (32.3)	42 (67.7)	1		
NUR	25 (41.0)	36 (59.0)	1.27	0.79–2.03	0.318
BSB	16 (29.6)	38 (70.4)	0.92	0.53–1.59	0.761
BDS	47 (54.0)	40 (46.0)	1.67	0.11–2.52	0.01
BPH	39 (35.4)	71 (64.6)	1.1	0.71–1.71	0.674
MBChB	190 (49.2)	196 (50.8)	1.52	1.05–2.22	0.027
**Year of study**					
I	48 (27.3)	128 (72.7)	1		
II	53 (35.8)	95 (64.2)	1.31	1.07–1.60	0.008
III	94 (50.5)	92 (49.5)	1.85	1.55–2.21	<0.001
IV	87 (59.2)	60 (40.8)	2.17	1.50–3.14	<0.001
V	55 (53.4)	48 (46.6)	1.95	1.68–2.28	<0.001
**Marital status**					
Married	32 (44.4)	40 (55.6)	1		
Single	276 (43.7)	355 (56.3)	0.98	0.74–1.31	0.914
Others*^1^	29 (50.8)	28 (49.2)	1.14	0.78–1.67	0.486
**Student sponsorship**					
Government	168 (40.6)	246 (59.4)	1		
Parents/guardian	137 (49.6)	139 (40.4)	1.22	1.02–1.46	0.027
Private sponsorship	28 (42.4)	38 (57.6)	1.04	0.82–1.33	0.718
Self	4 (100)	----	2.46	0.01–3.02	<0.001
**Student’s upkeep**					
Parents	193 (42.7)	259 (57.3)	1		
Guardian	25 (48.1)	27 (51.9)	1.12	0.77–1.64	0.538
Sponsor	13 (30.9)	29 (69.1)	0.72	0.49–1.06	0.097
Self	101 (51.3)	96 (48.7)	1.2	1.08–1.32	0.001
Spouse	5 (29.4)	12 (70.6)	0.68	0.02–2.27	0.541

**Table 3:** Multivariate analysis of the association between social-demographics and Hepatitis B vaccination status.

Variable	Prevalence ratio	95% CI	P-Value
Sex			
Female	1		
Male	0.79	0.69–0.91	0.004
**Course**			
BMR	1		
NUR	1.01	0.96–1.08	0.534
BSB	1.68	1.62–1.75	<0.001
BDS	2.56	2.43–2.70	<0.001
BPH	1.98	1.89–2.06	<0.001
MBChB	1.94	1.87–2.00	<0.001
**Year of study**			
I	1		
II	2.47	2.39–2.55	<0.001
III	3.28	3.20–3.35	<0.001
IV	2.07	1.46–2.93	<0.001
V	1.88	1.86–1.89	<0.001
**Student’s sponsorship**			
Government	1		
Parents/Guardian	1.28	0.99–1.65	0.059
Private	1.19	1.04–1.35	0.009
Self	2.08	1.57–2.74	<0.001

### Discussion

The proportion of students who were fully vaccinated was found to be 44.3% (95% CI; 35.2–53.4). Contrary to the Uganda Ministry of Health’s recommendation of 100% vaccination of students in the health-related field. This was consistent with findings by Atiba et al. (2014) in Nigeria where only 39.2% were fully vaccinated. This low coverage implies a low level of protection against Hepatitis B infection despite the potential risk to occupational exposure, which calls for intervention by policy makers.

The potential risk of occupational exposure to hepatitis B among these students was also high with about one third (36.7%) of the participants reporting an accidental needle stick injury since the beginning of their training. This is slightly lower than the findings in the Noubiap study (2013) among Cameroonian Medical students which demonstrated needle stick injury rate of 55.9%. Given the low vaccination completion and the needle stick injuries among the students in MakCHS, this could lead to continued transmission with eventual morbidity and mortality from Hepatitis B infection among students.

Among those who reported not being vaccinated, reasons for not receiving vaccination included high cost of vaccine (63%). Our finding differs from previous studies [7], [8] where availability was the most cited reason. This is probably because Hepatitis B vaccination service among the students at MakCHS is not free and there is no organized system for the students to attain this service. Consequently, more than half the participants were not vaccinated despite the potential risk of occupational exposure.

Most (67.8%) of the respondents perceived themselves at high risk of occupational exposure to HBV. The perception is consistent with the fact that the proportion of needle stick injuries was also high in our study. The variability in the risk perception could be arising from the differences in courses of the participants which in turn could explain the variability in their vaccination status as well.

Male students were about 20% less likely to be vaccinated, with a Prevalence Ratio of 0.79 (p-value 0.001). This was consistent with findings from the study by Atiba et al. (2014) where females had higher odds of being vaccinated. Probably because generally females are better health seekers than males [9]. This means that any interventions designed to enhance uptake of intervention programs like vaccination against HBV among students should be gender sensitive, in terms of involvement especially to male students.

BDS students had the highest likelihood of being vaccinated with a prevalence ratio of 2.56 (p-value <0.001) followed by BPH, MBChB and BSB compared to BMR. NUR was not significantly associated with vaccination. This is possibly due to group influence and the difference in risk perception and knowledge. The findings highlight the need for targeted interventions for the different subgroups of students.

Year of study was also significantly associated with vaccination. Students in Year III which is also a clinical year for most courses, were most likely to be vaccinated when compared to those in Year I, with a prevalence ratio of 3.28 (p-value <0.001) This is probably due to group influence and the wave of media influence that followed the launch of the statutory instrument by Ministry of Health declaring mandatory vaccination of health workers in 2014. There was significant interaction between year of study and course.

Student’s sponsorship was also a significantly associated with vaccination. Privately sponsored students as well those who sponsored themselves were most likely to report being vaccinated compared to government sponsored students. Probably these students are provided sufficient pocket money, or they are self-reliant financially to meet the students’ needs including healthcare needs like vaccination.

Most participants (74.6%) were knowledgeable about Hepatitis B infection. However, there was no association between knowledge category on hepatitis B and hepatitis B vaccination status. Our finding differs from previous studies [10], [6] which reported high odds of vaccination among knowledgeable students. It therefore suffices that increasing knowledge among the students in MakCHS is not what should be prioritized to improve the vaccination coverage but rather ensuring that Hepatitis B vaccines are available and affordable.

### Strength of the study

The large sample size used for assessment of the vaccination status was adequate, which minimized the possibility of random error. Also, the data collection tool used was pre-tested and standardized before being administered which supports the validity of the study findings.

### Conclusion

The low coverage coupled with a high prevalence of needle prick injuries raises a safety concern that needs attention by the policy makers. The main reason for low coverage were high cost of the vaccine, which calls for free vaccination service.

## Limitations

Vaccination status was assessed based on self-report rather than Antibody to hepatitis B surface antigen (anti-HBs) status, which could have biased the outcome as some people may have failed to recall their vaccination history or some may have given false information on the doses they received. The proportion of those deemed to be fully protected could be different if assessed based on anti-HBs levels. The other limitation was a low turn up of students which forced us to enroll everyone who fulfilled the eligibility criteria. It is possible that students that were interviewed may have been different from those that we missed, hence a possible bias in our estimates.

## Supporting information

S1 File This is the questionaire. (DOCX)

## Abbreviations

Anti-HBs: Antibody to hepatitis B surface antigen
BDS: Bachelor of Dental Surgery
BEH: Bachelor of Environmental Health Science
BMR: Bachelor of Science in Medical Radiography
BPH: Bachelor of Pharmacy
BSB: Bachelor of Science in Biomedical Sciences
HBsAG: Hepatitis B surface antigen
HBV: Hepatitis B Virus
MakCHS: Makerere University College of Health Sciences
MBChB: Bachelor of Medicine and Bachelor of Surgery
NUR: Bachelor of Science in Nursing
PEP: Post Exposure Prophylaxis
PPE: Personal Protective Equipment
PR: Prevalence ratio
SOMREC: School of Medicine Research Ethics Committee
WHO: World Health Organization
