# Supplementary material for: Hepatitis B vaccination status and associated factors among undergraduate students of Makerere University College of Health Sciences
Source: PLoS One. 2019 Apr 5;14(4):e0214732. doi: 10.1371/journal.pone.0214732 (PMC6450609; doi:10.1371/journal.pone.0214732)
Supplement: S1 File — (DOCX) [file pone.0214732.s001.docx]

**APPENDIX II DATA COLLECTION**

**DATA COLLECTION TOOL**

**STUDY*:*** *HEPATITIS B VACCINATION STATUS AND ASSOCIATED FACTORS AMONG UNDERGRADUATE STUDENTS OF MAKERERE UNIVERSITY COLLEGE OF HEALTH SCIENCES*

**RESEARCHER:** *DR YVETTE WIBABARA, MBChB (MAK)*

**DATE OF DATA COLLECTION (DD/MM/YYYY) ………………….**

**INSTRUCTIONS TO STUDY PARTICIPANTS:**

**(Please read before completing questionnaire): Having received information about this study, you can now fill the questionnaire to participate. If you do not wish to complete the questionnaire, please keep the blank version and I will collect it with all other questionnaires.**

**Please only complete the questionnaire if you are offering (MBCHB), (NUR), (BDS), (BMR) (BPH) or (BSB)**

**Note:** This questionnaire is anonymous; please do not write your name on it. Kindly give answers to all the questions as it pertains to you and please answer as truthfully as you can.

Please check (✔) only the box that most correctly answers the question, making sure you make only one selection for each question except where otherwise indicated.

**NB: If you have ever been diagnosed with Hepatitis B infection, kindly do not fill this questionnaire. Just check (✔) in this box and give the questionnaire back to the research assistant**. **Also provide your contact here……………………………………………………... our research team will get in touch with you for more information,**

**Section A: Demographic Questions**

**QN1**. What is your sex? 1. Female 2. Male

**QN 2**. How old were you on your last birthday?

**QN 3**. What course are you offering? 1. MBChB 2. BPH 3. BDS

4. BSB 5. NUR 6. BMR

**QN 4**. Which year are you? I II III IV V

**QN 5**. What is your marital status? 1. Married 2. Single 3. Others

**QN 6**. What is your religion 1. Christian 2. Moslem 3. Others

**QN 7.** Which sponsorship scheme are you on? 1. Government 2. Private(parents/guardian)

3. Other private sponsorship

**QN 8**. Who is taking care of your upkeep while at the university?

1. Parents 2. Guardian 3. Sponsor 4. Self 5. others

**QN 9**. What is your nationality? 1. Ugandan 2. Non-Ugandan

If the answer to the above question is Non-Ugandan, go to **question 11**

**QN 10**. What region of Uganda do you come from? 1. Central 3. Northern

2. Western 4. Eastern

**QN 11.** What is your country of origin? **(For Non-Ugandans)**

............................................................

**Section B. Questions on knowledge of Hepatitis B infection**

**QN 12.** Have you ever heard about hepatitis B? 1. No 2. Yes

**QN 13**. Is hepatitis B infection preventable? 1. No 2. Yes 3. I don’t know

**QN 14**. What is the mode of spread of HBV? 1. No 2. Yes

|  |  |
| --- | --- |
|  |  |
|  |  |
|  |  |
|  |  |

Tick all that apply a. Transfusion with infected blood

b. Unprotected sex

c. Sharing sharp instruments

f. Mother to child during pregnancy

g. Sharing food with an infected person

1. No 2. Yes

|  |  |
| --- | --- |
|  |  |
|  |  |

**QN 15**. Which of these is a complication of hepatitis B? a. Liver cirrhosis

Tick all that apply b. Liver cancer

c. None of the above

**QN 16.** Is there post exposure prophylaxis for HBV? 1. No 2. Yes 3. I don’t know

**QN 17**. Have you ever been screened for hepatitis B? 1. No 2. Yes

Knowledge score………………………**… (Please do not fill this part)**

**Section C Questions related to HBV vaccination status**

**QN 18**. Have you ever been vaccinated against hepatitis B?

1. No 2. Yes 3. Not sure

If the answer to the above question is No, **go to question 23**

**QN 19.** When did you receive the first dose?

1. About one month ago 2. Between one month and Six months ago

3. > Six months ago 4. Not sure

**QN 20**. How many doses have you received so far?

1. One 2. Two 3. Three 4. >three 5. Not sure

**QN 21**. What was your reason for getting vaccinated? (Tick the most appropriate)

1. I fear getting Hepatitis B

2. It is a requirement by Ministry of Health and the University

3. All my friends were doing it

**QN 22**. Do you have a vaccination card? 1. Yes 2. No

**QN 23**. What is the reason for not getting vaccinated? **(For those who are not vaccinated)**

1. I am not at risk

2. The vaccine is expensive

3. I don’t know where to find the vaccine

**QN 24**. How many doses of HBV vaccine are required for complete protection?

1. One dose 2. Two doses 3. Three doses 4. Not sure

**QN 25**. Do you think you are at risk of occupational exposure to hepatitis B virus?

1. No risk of exposure

2. Low risk of exposure

3. High risk of exposure

4. Not sure

**QN 26**. Have you ever had a needle stick injury? 1. No 2. Yes

If the answer to the above question is YES, go to question 27 and 28

**QN 27.** Did you take any post exposure prophylaxis (PEP) for HIV?

1. No 2. Yes 3. It was not necessary

**QN 28.** Did you take any post exposure prophylaxis (PEP) for Hepatitis B?

1. No 2. Yes 3. It was not necessary

4. It was necessary but not available

Thank you very much for participating in this study. If you have any additional thoughts, questions, or comments, please feel free to write them on any blank page.

Please return this questionnaire to the research assistant. If you would like to receive a copy of the final analysis or if you have questions or comments, please contact the researcher on +256773523815 or send an email to [yvettebarya@gmail.com](mailto:yvettebarya@gmail.com)
